# Validity and reliability of self-reported methods for assessment of 24-h movement behaviours: a systematic review

**DOI:** 10.1186/s12966-024-01632-4

**Published:** 2024-08-02

**Authors:** Anja Šuc, Lea Einfalt, Nejc Šarabon, Kaja Kastelic

**Affiliations:** 1 Faculty of Health Sciences, University of Primorska, Izola, Slovenia; 2 InnoRenew CoE, Izola, Slovenia; 3 Andrej Marušič Institute, University of Primorska, Koper, Slovenia

**Keywords:** Time-use epidemiology, Measurement, Physical activity, Sedentary behaviour, Sleep, Physical behaviours, Time-use questionnaire, Time-use recall, Time-use diary

## Abstract

**Background:**

Time spent in sleep, sedentary behaviour (SB), and physical activity are exhaustive and mutually exclusive parts of a 24-h day that need to be considered in a combination. The aim of this study was to identify validated self-reported tools for assessment of movement behaviours across the whole 24-h day, and to review their attributes and measurement properties.

**Methods:**

The databases PubMed, Scopus, and SPORTDiscus were searched until September 2023. Inclusion criteria were: (i) published in English language, (ii) per-reviewed paper, (iii) assessment of self-reported time spent in sleep, SB, and physical activity, (iv) evaluation of measurement properties of all estimates across the full 24-h day, and (v) inclusion of adolescents, adults, or older adults. The methodological quality of included studies was assessed using the Consensus-based Standards for the selection of health Measurement Instruments checklist.

**Results:**

Our search returned 2064 records. After studies selection, we included 16 articles that reported construct validity and/or test-retest reliability of 12 unique self-reported tools – eight questionnaires, three time-use recalls, and one time-use diary. Most tools enable assessment of time spent in sleep, and domain-specific SB and physical activity, and account that sum of behaviours should be 24 h. Validity (and reliability) correlation coefficients for sleep ranged between 0.22 and 0.69 (0.41 and 0.92), for SB between 0.06 and 0.57 (0.33 and 0.91), for light-intensity physical activity between 0.18 and 0.46 (0.55 and 0.94), and for moderate- to vigorous-intensity physical activity between 0.38 and 0.56 (0.59 and 0.94). The quality of included studies being mostly fair-to-good.

**Conclusions:**

This review found that only a limited number of validated self-reported tools for assessment of 24-h movement behaviours are currently available. Validity and reliability of most tools are generally adequate to be used in epidemiological studies and population surveillance, while little is known about adequacy for individual level assessments and responsiveness to behavioural change. To further support research, policy, and practice, there is a need to develop new tools that resonate with the emerging 24-h movement paradigm and to evaluate measurement properties by using compositional data analysis.

**Systematic review registration:**

PROSPERO CRD42022330868.

**Supplementary Information:**

The online version contains supplementary material available at 10.1186/s12966-024-01632-4.

## Background

Sleep, sedentary behaviour (SB), and physical activity (i.e., 24-h movement behaviours) are important determinants of health and well-being [1, 2]. Research shows that sufficient sleep duration, less SB, and greater physical activity are associated with a decreased risk of numerous chronic non-communicable diseases including cardiovascular disease, type 2 diabetes, cancer, mental disorders, and all-cause mortality [2–5].

Movement behaviours have been traditionally examined and promoted in isolation from each other. However, a recent recognition that time spent in sleep, SB, and physical activity are exhaustive and mutually exclusive parts of any time period (e.g., 24-h day) has shifted the paradigm towards examining movement behaviours in a combination [6–8]. Moreover, particular concern has been drawn to the methodological shortcomings of most previous epidemiological studies on time spent in movement behaviours that examined specific movement behaviour in isolation while violating the assumptions of statistical methods used [9, 10]. Data quantifying time spent in movement behaviours are specific type of data (i.e., compositional data), and their specific mathematical properties need to be respected by using sound statistical methods (i.e., compositional data analysis). In compositional data, relevant information is in the relative distribution of the components, which indicates that the components need to be examined in a combination [9–11]. Therefore, there is a need for research tools that simultaneously assess movement behaviours across the whole 24-h day.

This novel paradigm has already been adopted by some public health authorities who also recognised the importance of promoting healthy movement behaviours in an integrated way and developed 24-h movement guidelines [12–19]. According to such guidelines, it is recommended to engage in moderate- to vigorous-intensity physical activity (MVPA) for at least 150 min per week, in light-intensity physical activity (LPA) for several hours per day, to avoid SB to the extent that total daily duration do not exceed eight hours per day, while getting between seven and nine hours of sleep. To monitor population prevalence and trends of adherence to the novel 24-h movement guidelines, surveillance systems need to be adapted accordingly [20].

The assessment of 24-h movement behaviours is also needed for individual level counselling, prescription, and referral of guiding discussions regarding behavioural change. Such treatments could be conducted in clinical care settings and community programs for healthy lifestyle promotion, disease prevention and management as well as in occupational and school settings. It has been advocated that integrated 24-h movement paradigm cater to individual differences (e.g., physical abilities, preferences) and offer a wide variety of counselling options on behavioural change (e.g., trading SB for LPA and/or MVPA only, while keeping sleep unchanged), that can bring health benefits [18]. However, a recent scoping review on features, perceptions, and effectiveness of tools to guide discussions on physical activity, SB, and/or sleep between health care providers and patients showed that tools to guide discussions on integrated 24-h movement behaviours are lacking [21].

Therefore, simultaneous assessment of sleep, SB, and physical activity is needed for research, policy, and practice. Such assessment can be conducted using device-based methods (e.g., accelerometers, inclinometers), self-reported methods (e.g., questionnaires, diaries), or using a combination of both methods (e.g., using sleep time diary to inform sleep detection algorithms for accelerometer data [22]). Both groups of measurement methods show certain strengths and weaknesses; and the choice of the measurement tool is usually guided by the level of reliability and validity required for specific purpose of use, resources available, feasibility, practicality, acceptability, sustainability, and the need to provide immediate feedback [23–27]. While device-based methods have advantages of providing more valid estimates, self-reported methods present lower costs, lower burden, and higher compliance. Self-reported methods can also provide contextual information on movement behaviours (e.g., where, with whom) and estimates of movement behaviours from more distant past. Self-reported tools are therefore indispensable in large-scale epidemiological studies, population surveillance, and practice [21, 26, 28].

Most of the self-reported tools were developed for assessment of only one or two movement behaviours [29–38], and to the best of our knowledge, self-reported tools for assessment of overall 24-h movement behaviours are scarce. While sleep, SB, and physical activity can be assessed using a combination of different self-reports, such an approach might be compromised and/or being inconvenient as different self-reports may have different recall periods, administration guidelines, or instructions to complete the items. It is also less likely that the sum of all movement behaviours assessed using different tools would equal 24 h (or other finite total). This might be of particular concern when using compositional data analysis that closes composition to the finite total [11] and proportionally rescale the components that do not add to the finite total (e.g., 24 h). Rescaling the data is likely to change measurement properties that need re-evaluation. Therefore, the aim of this study was to identify validated self-reported tools for assessment of movement behaviours across the whole 24-h day, and to review their attributes (movement behaviours being assessed including temporal and contextual information, accounting for a 24-h day, recall period, number of questions) and quantitative measurement properties (construct validity, test-retest reliability, responsiveness).

## Methods

This systematic review was conducted in accordance with the Preferred Reporting Items for Systematic Reviews and Meta-Analyses (PRISMA) statement [39], and it was registered in the International Prospective Register of Systematic Reviews (PROSPERO) with a registration number CRD42022330868. The review protocol can be accessed on the PROSPERO website (https://www.crd.york.ac.uk/prospero/).

### Eligibility criteria

We included studies that met the following criteria: (i) published in English language, (ii) published in a peer-reviewed journal, (iii) reported assessment of time spent in sleep, SB, and physical activity using a single self-reported tool (without any restriction regarding the mode of administration), (iv) reported construct validity (i.e., the extent to which an instrument provides comparable measures to other validated instrument that measure the construct of interest [40]), test-retest reliability (i.e., the extent to which an instrument provide measures that are consistent from one test administration to the next [40]), or responsiveness (i.e., the ability of an instrument to detect change over time in the construct to be measured [40]) of self-reported estimates of movement behaviours across the full 24-h day, and (v) included adolescents (aged 12 to 17 years), adults (aged 18 to 64 years), or older adults (aged 65 years and older). No limitations regarding the sample size and health status of participants were applied. We excluded studies that reported validity by comparing measures of different constructs (e.g., comparing self-reported MVPA with physical fitness test score), secondary data analysis studies, reviews, and meta-analysis.

### Literature search and study selection

A literature search was performed in databases of PubMed, Scopus, and SPORTDiscus. The primary search query combined terms: movement behaviours, self-reported method, and validity/reliability (Supplementary Table 1). The search with no publication time limits was performed in May 2022, and updated in September 2023.

All hits from the databases were transferred to the Mendeley Desktop Reference Management Program. After removing the duplicates, three authors (AŠ, LE, and KK) independently screened the titles and abstracts for eligibility. Afterwards, two authors (AŠ or LE, and KK) independently screened the full texts of potentially relevant articles for the final decision on study inclusion. Disagreements between authors were resolved through discussion and consensus. If there were any uncertainties, the fourth author (NŠ) was consulted. Additionally, to identify any relevant articles that might be missed by our primary search query, we performed a backward and forward citation searching, screened relevant reviews and meta-analysis that were identified through primary search query, and authors’ archive of references. Also, we conducted a secondary search that combined terms: title of the tool (tools that were identified during primary search) and validity/reliability.

### Data extraction

Data from the included studies were extracted by two authors (AŠ or LE) and checked by the third author (KK). Disagreements between authors were resolved through discussion and consensus. If there were any uncertainties, the fourth author (NŠ) was consulted. The following information were extracted: (i) first author, (ii) year of publication, (iii) title of the self-reported tool, (iv) type of the self-reported tool, (v) movement behaviours assessed using self-reported tool, (vi) whether and how self-reported tool accounted for the finite sum of daily time spent in sleep, SB, and physical activity, (vii) number of questions, (viii) recall period, (ix) sample characteristics (i.e., sample size, proportion of females, mean age), (x) language of evaluated self-reported tool, (xi) reference tool used, (xii) time interval between two administrations, (xiii) construct validity indicators, (xiv) test-retest reliability indicators, and (xv) responsiveness indicators.

### Quality assessment

The methodological quality of included studies was assessed using the Consensus-based Standards for the selection of health Measurement Instruments (COSMIN) checklist [41]. This checklist assesses the appropriateness of study design and statistical methods used in individual studies on measurement properties. The quality of the studies for evaluating validity and reliability was assessed using the 4-point scale (i.e., excellent, good, fair, poor) for each of the checklist items, while the final quality score was assessed using the “worst score counts” principle [42]. The COSMIN checklist used is available in Supplementary Tables 2 and 3. The quality assessment was done independently by two authors (AŠ or LE, and KK). Disagreements between authors were resolved through discussion and consensus. If there were any uncertainties, the fourth author (NŠ) was consulted.

### Data presentation and interpretation

The data were narratively presented in tables and arranged according to the type of self-reported method (i.e., questionnaires, time-use recalls, time-use diary). Self-reported tools were listed alphabetically. The first table contains data on attributes of self-reported tools, the second table contains data on construct validity, and the third table contains data on test-retest reliability of self-reported tools.

Criteria on interpreting construct validity and test-retest reliability correlation coefficients were set a priori and were based on the findings from previous systematic reviews on measurement properties of physical activity and SB self-reports (Spearman/Pearson correlation coefficients for construct validity usually range from approximately 0.30 to 0.50 [29, 32, 34]; and Intraclass correlation coefficients (ICC) for test-retest reliability usually range from approximately 0.50 to 0.80 [29, 32, 34]). Convergent validity correlation coefficients were interpreted as: 0 to 0.20 as poor; 0.21 to 0.40 as fair; 0.41 to 0.60 as moderate; 0.61 to 0.80 as substantial; 0.81 to 1.00 as nearly perfect [43]. Test-retest reliability correlation coefficients were interpreted as: 0 to 0.49 as poor; 0.50 to 0.74 as moderate; 0.75 to 0.89 as substantial; 0.90 to 1.00 as nearly perfect [44].

## Results

**Fig. 1:**
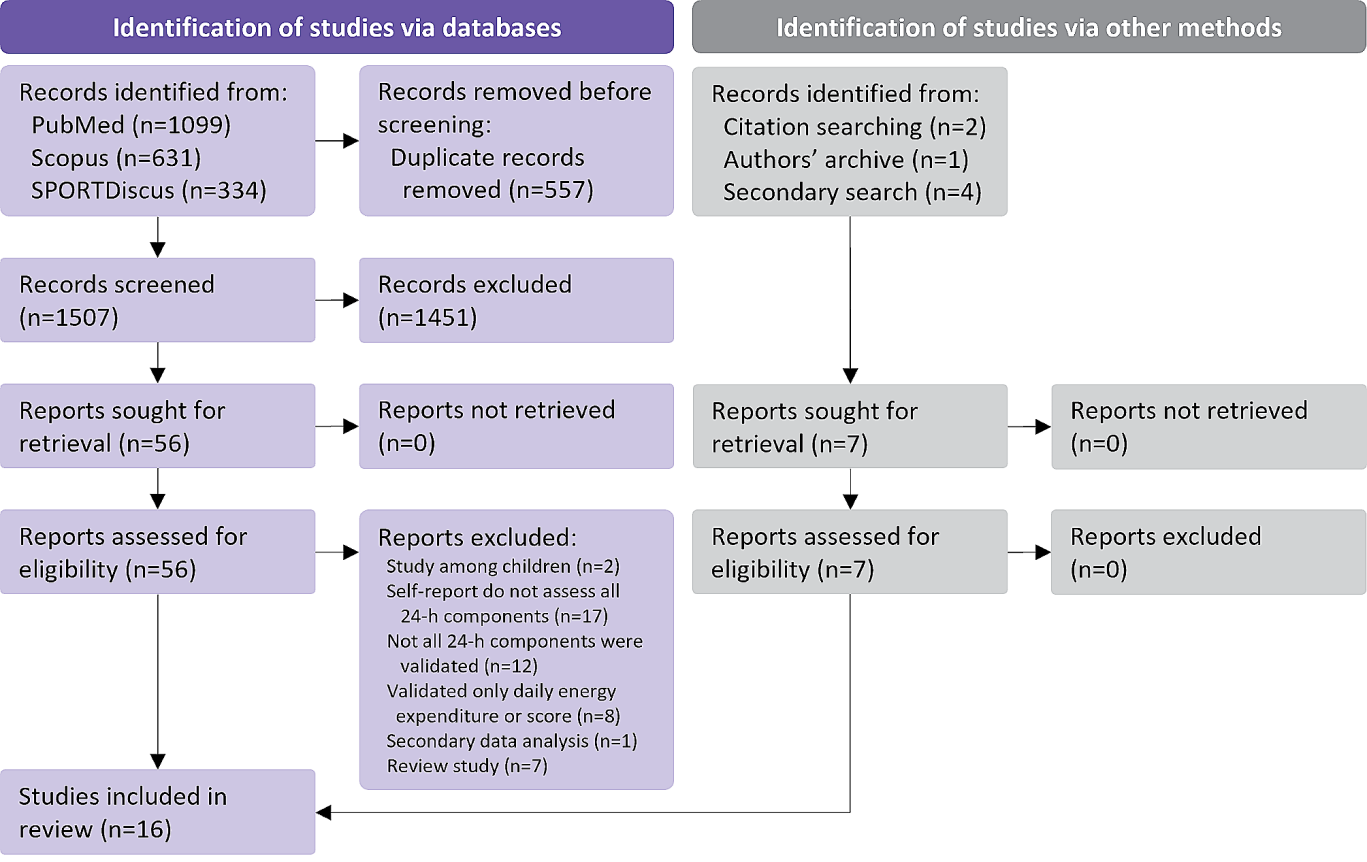
PRISMA flow diagram on the systematic search process

Our search query returned 2064 records (Fig. 1). After removing the duplicates, we screened titles and abstracts of 1507 records. We identified 56 potentially relevant articles and assessed their full texts for eligibility. Of these, we excluded 2 articles that included only children aged 11 years or less [45, 46], 17 articles on tools that do not assess all components across the full 24-h day [47–63], 12 articles that reported measurement properties of some but not all components across the full 24-h day [64–75], 7 articles that reported only measurement property of assessing total daily energy expenditure [76–82] and integrated movement behaviours score [83], 1 article that reported secondary data analysis [84], and 7 review articles [21, 38, 85–89]. Additional seven records were identified through other sources. Finally, 16 articles that reported measurement properties of 12 unique self-reported tools were included in our review [90–105]. There was 100% agreement on the selection of all 16 included articles between the two reviewers (AŠ or LE, and KK).

### Attributes of self-reported methods

We identified eight unique self-reported questionnaires, three time-use recalls, and one time-use diary (Table 1). Most questionnaires ask about sleep, and domain-specific SB and physical activity, and account that their sum should be 24 h. The latter is achieved by subtracting non-SB estimates from 24 h to obtain SB (Simple Physical Activity Questionnaire [SIMPAQ]) or by assigning the “remaining time to 24 hours” to SB (Japan Public Health Center-based prospective study- physical activity questionnaire [JPHC-PAQ], Physical activity questionnaire [PAQ], Sedentary Time and Activity Reporting Questionnaire [STAR-Q]) or LPA (Daily Activity Behaviours Questionnaire [DABQ], Physical Activity Scale 2 [PAS 2]). The same method of accounting for 24-h day is also used in one time-use recall (7-Day Physical Activity Recall [7D PAR]), while other two time-use recalls (Computer-Based 24-Hour Physical Activity Recall [cpar24], Multimedia Activity Recall for Children and Adults [MARCA]) are computerised and features of the program ensure/facilitate complete data entry (i.e., 24 h of activities). In computerised time-use recalls, a responder is asked to report activities in the order that they were performed during the day by choosing from a custom compendium of activities. Similarly, in time-use diary (Time-use diary from the Harmonised European Time Use Study [TUD HETUS]) a responder is asked to record activities (in their own words) in the order that they were performed during the day. In computerised time-use recalls and in time-use diary, reported daily activities are converted into sleep, SB, and physical activity by using a compendium of physical activities.

**Table 1:** Attributes of self-reported measurement tools

Title of the tool	Movement behaviours assessed	Temporal and contextual information	Account for a 24-h day	Recall period	Number of questions
**Questionnaires**					
Daily Activity Behaviours Questionnaire (DABQ) Kastelic et al., [94], [93]	Sleep, occupational activity (SB, LPA, MVPA), commuting activity (SB, LPA, MVPA), and other non-occupational activity (SB, LPA, MVPA), including walking, sport participation, screen time	Sleep onset and offset (hh: mm), start/end of occupational time (hh: mm), weekday/weekend; domain-specific SB and PA	Yes (the remaining time to 24 h is assigned as LPA; if reported domain-specific movement behaviours exceed time spent in corresponding domain then behaviours within that domain are proportionally downscaled)	Past week	32
Japan Public Health Center-based prospective study- physical activity questionnaire (JPHC-PAQ) Kikucki et al., [95]	Sleep, occupational and household activity, including transportation (sitting, standing, walking, strenuous work), leisure-time activities (walking slowly, walking quickly, light to moderate and strenuous exercise)	Domain-specific SB and PA	Yes (the remaining time to 24 h is assigned as 1.3 METs, i.e., SB)	Past year	4
Physical activity questionnaire (PAQ) Bharathi et al., [99]	Sleep, occupational activity (sitting, standing, walking, more strenuous activities), discretionary activity (sports and games, hobbies, household chores, discretionary sedentary activity)	Domain-specific SB and PA	Yes (the remaining time to 24 h is assigned as 1.2 METs, i.e., SB)	Past month	5
Physical Activity Questionnaire from the Southern Community Cohort Study (PAQ SCCS) Buchowski et al., [90]	Sleep, SB (car or bus, sitting at work, viewing TV, or seeing movies, using a computer at home, other sitting activities), physically active behaviours (light, moderate, vigorous occupational/household work, sport/exercise, slow walking, fast walking)	Domain-specific SB and PA	Not reported	Unanchored day	Not available
Physical Activity Scale 2 (PAS 2) Pedersen et al., [101]	Sleep, occupational activity (sitting, standing/walking, heavy physical work), active commuting, leisure activity (SB, LPA, MPA, VPA)	Domain-specific SB and PA	Yes (the remaining time to 24 h is assigned as LPA; if total time exceed 24 h then the surplus hours are subtracted from LPA)	Unanchored week	9
Physical Activity Scale 2.1 (PAS 2.1) - modified (transportation sitting item was added) Valles-Medina et al., [104]	Sleep, work/school hours activity (sitting, standing/walking, climbing stairs/carrying heavy objects), transportation sitting, leisure activity (sitting while using screens/reading, LPA, MPA, VPA)	Domain-specific SB and PA	Not reported	Past week	9
Sedentary Time and Activity Reporting Questionnaire (STAR-Q) Csizmadi et al., [91]	Sleep, physical activities, and sedentary behaviours across all domains: eating, personal/medical care, occupation, transportation, household, yard work, caregiving, exercise, light leisure (e.g., television-watching, personal computer time, reading, hobbies, etc.), stair-climbing, “other” activities	Domain-specific SB and PA; social context for some activities (e.g., caring for children, elderly or dependent adult), physical context for some activities (e.g., yard work)	Yes (the remaining time to 24 h is assigned as 1.2 METs, i.e., SB)	Past month	88
Simple Physical Activity Questionnaire (SIMPAQ) Rosenbaum et al., [102]; Vancampfort et al., [105]	Time in bed, napping, SB, walking, exercise, non-structured PA	Time in bed onset and offset (hh: mm)	Yes (only when using alternative method to calculate SB: SB = 24 h - PA - time in bed)	Past week	8
Simple Physical Activity Questionnaire (SIMPAQ) - modified (standing item was added) Schilling et al., [103]	Time in bed, napping, SB, standing, walking, exercise, non-structured PA	Time in bed onset and offset (hh: mm)	Yes (only when using alternative method to calculate SB: SB = 24 h - PA - time in bed)	Past week	9
24-hour movement behaviors questionnaire (24HMBQ) Zheng et al., [98]	Sleep, SB (study, screen time, other SB), LPA (exercise, transport, dormitory life), MPA (exercise, transport, dormitory life), VPA (exercise, transport, dormitory life)	Time in bed onset (hh: mm), sleep offset (hh: mm), weekday/weekend; domain-specific SB and PA	Not reported	Past week	33
**Time-use recalls**					
7-Day Physical Activity Recall (7D PAR) - modified (sitting item was added) Welk et al., [97]	Sleep, sitting, LPA, MPA, VPA, VVPA	Time of day for PA (i.e., morning, afternoon, evening)	Yes (the remaining time to 24 h is assigned as LPA)	Past week	Report activities in a segmented 7-day format (morning, afternoon, evening), for each physical activity longer than 10 min, responder is asked to rate the intensity as moderate, hard, or very hard; a responder is also asked about time spent sleeping and sitting for each day separately
Computer-Based 24-Hour Physical Activity Recall (cpar24) Kohler et al., [96]	Daily activities within the “super domains”: sleeping and reclining, personal care, food preparation and eating, walking, transportation, and traveling, household chores, occupational activity, shopping, errands, and appointments, leisure and hobbies, sports, family life and social activities, outdoor activities, lawn and garden, miscellaneous activities	Reporting specific activities chronologically across the day (start and end times); social context for some activities (e.g., family life and social activities), physical context for some activities (e.g., outdoor activities)	Yes (to ensure complete data entry, respondent is informed about missing or incomplete activity entries (i.e., time gaps) with the option of adding new activity items to arrive at the desired total amount of 24 h of logged activities per day)	Past day	Report activities in the order that they were performed in time slices of 5 min or more, by choosing from a custom compendium of 262 activities; for some activities, additional entries about the intensity and posture are required to be reported
Multimedia Activity Recall for Children and Adults (MARCA) Hunt et al., [100]	Daily activities within the “super domains”: physical activity, screen time, chores, work/study, sociocultural, self-care, transport, sleep, quiet time	Reporting activities chronologically across the day (start and end times); social context for some activities (e.g., social activities), physical context for some activities (e.g., gardening)	Yes (features of the programme allow for valid data entry, e.g., not allowing large gaps of missing time, or overlapping activities to be reported)	Past 2 days	Report activities in the order that they were performed in time slices of 5 min or more, by choosing from a custom compendium of over 520 activities, additional entries about the intensity for some activities
**Time-use diary**					
Time-use diary from the Harmonised European Time Use Study (TUD HETUS) Harms et al., [92]	The diary is completed in respondent’s own words	Reporting activities chronologically across the day (start and end times); reporting social (e.g., alone, partner) and physical context (e.g., at home, in store) for all reported activities; reporting enjoyment while engaging in activities	Yes (the diary has pre-defined recording fields that cover 24-h period)	Current day	The diary covers 24 h in 10 min intervals, and has six types of recording fields: primary and (up to three simultaneous) secondary activities (free text), co-presence, location/travel mode, technology use, and enjoyment (pre-coded)

*Note:* SB, sedentary behaviour; PA, physical activity; LPA, light-intensity physical activity; MET, metabolic equivalent of task; MPA, moderate-intensity physical activity; MVPA, moderate- to vigorous-intensity physical activity; VPA, vigorous-intensity physical activity; VVPA, very vigorous-intensity physical activity

Self-reported measurement tools differ substantially regarding recall period (ranging from the past day to the past year) and comprehensiveness (number of questions for questionnaires ranging from 4 to 88, while time-use recalls and time-use diary record activities over the past day to the past week). Most questionnaires assess total sleep time, domain-specific SB, and domain- and intensity-specific physical activity (DABQ, JPHC-PAQ, PAQ, PAQ SCCS, PAS 2, STAR-Q, 24HMBQ). Most questionnaires also assess at least some specific types of SB and physical activity (Table 1), while only some questionnaires assess sleep timing (DABQ, SIMPAQ, 24HMBQ), movement behaviours at weekdays/weekend days separately (DABQ, 24HMBQ), and social and physical context for some activities (STAR-Q). Time-use recalls (cpar24, MARCA) and time-use diary (TUD HETUS) provide detailed data on specific types of activities and the timing of activities performed during the 24-h day. The TUD HETUS also provide a social and physical context for all reported activities and the level of enjoyment while engaging in activities.

### Validity of self-reported methods

A total of 11 studies evaluated validity of 10 self-reported tools for assessment of 24-h movement behaviours among adults (Table 2). Two studies were ranked with an excellent quality [92, 94], three with a good quality [93, 101, 103], three with a fair quality [91, 95, 96], and three with a poor quality [90, 97, 98]. Device-based method was used as a reference method in seven studies, while four studies used another self-reported method to evaluate validity. All but one study used Pearson/Spearman’s correlation coefficient between self-reported method and reference method. Some studies also reported Intraclass correlation coefficient and/or Bland-Altman statistics (e.g., mean difference, limits of agreement).

**Table 2:** Validity of self-reported measurement tools

Title of the tool	Language	Sample characteristics	Reference method used	Validity indicators	Quality of the study
**Questionnaires**					
Daily Activity Behaviours Questionnaire (DABQ) Kastelic et al., [94]	Slovenian version	Adults (employed) *n* = 107 (45 female) age = 39 ± 8 years	activPAL accelerometer (thigh worn) + sleep diary	Sleep: rho = 0.66 (95% CI: 0.54, 0.76); ICC = 0.63 (95% CI: 0.52, 0.71); mean difference (DABQ - Acc) = 1 min/day (95% CI: -8.7, 10.8); 95% LoA: -98.7 to 100.8 min/day SB: rho = 0.42 (95% CI: 0.25, 0.56); ICC = 0.29 (95% CI: 0.09, 0.45); mean difference (DABQ - Acc) = -96.5 min/day (95% CI: -124.8, -68.1); 95% LoA = -386.5 to 193.6 min/day LPA: rho = 0.45 (95% CI: 0.29, 0.59); ICC = 0.22 (95% CI: -0.00, 0.41); mean difference (DABQ - Acc) = 134.5 min/day (95% CI: 106.8, 162.3); 95% LoA = -149.2 to 418.2 min/day MVPA: rho = 0.38 (95% CI: 0.21, 0.53); ICC = 0.24 (95% CI: -0.05, 0.48); mean difference (DABQ - Acc) = -39.1 min/day (95% CI: -44.7, -33.5); 95% LoA = -96.3 to 18.1 min/day	Excellent
Daily Activity Behaviours Questionnaire (DABQ) Kastelic et al., [93]	German version	Older adults (retired) *n* = 77 (45 female) age = 68 ± 5 years	activPAL accelerometer (thigh worn) + sleep diary	Sleep: rho = 0.69 (95% CI: 0.55, 0.79; *p* < 0.001); mean difference (DABQ - Acc) = 8 min/day (95% CI: -6, 22); 95% LoA: -111.1 to 127 min/day SB: rho = 0.35 (95% CI: 0.14, 0.53; *p* < 0.01); mean difference (DABQ - Acc) = -135 min/day (95% CI: -174, -97); 95% LoA: -466.8 to 196 min/day LPA: rho = 0.24 (95% CI: 0.02, 0.44; *p* < 0.05), mean difference (DABQ - Acc) = 141 min/day (95% CI: 104, 178); 95% LoA: -177.1 to 458.5 min/day MVPA: rho = 0.52 (95% CI: 0.33, 0.66; *p* < 0.001), mean difference (DABQ - Acc) = -13 min/day (95% CI: -25, - 2); 95% LoA: -112.3 to 85.9 min/day	Good
Japan Public Health Center-based prospective study- physical activity questionnaire (JPHC-PAQ) Kikucki et al., [95]	Japan version	Adults & older adults (mostly employed) *n* = 110 (57 female) age = 61 ± 6 years	24-h Activity Record	Sleep: rho = 0.398 (*p* < 0.001) SB + LPA: rho = 0.581 (*p* < 0.001) MPA: rho = 0.345 (*p* < 0.001) VPA: rho = -0.093 (*p* = 0.336) MVPA: rho = 0.563 (*p* < 0.001)	Fair
Physical Activity Questionnaire from the Southern Community Cohort Study (PAQ SCCS) Buchowski et al., [90]	English version	Adults *n* = 112 for T1 *n* = 86 for T2 Characteristics of baseline sample (87 African American, 31 non-Hispanic whites): *n* = 118 (61 female) age = 55 ± 8 years	Last Month Physical Activity Survey (LMPAS) + sleep item	Whites Sleep: rho = 0.59 (*p* = 0.005) for T1, rho = 0.49 (*p* = 0.02) for T2 SB: rho = 0.57 (*p* = 0.01) for T1, rho = 0.51 (*p* = 0.02) for T2 Occupational/household LPA: rho = 0.54 (*p* = 0.01) for T1, rho = 0.30 (*p* = 0.19) for T2 Occupational/household MPA: rho = 0.48 (*p* = 0.03) for T1, rho = -0.06 (*p* = 0.80) for T2 Occupational/household VPA: rho = 0.14 (*p* = 0.50) for T1, rho = 0.22 (*p* = 0.34) for T2 EE of sport/exercise: rho = 0.35 (*p* = 0.10) for T1, rho = 0.39 (*p* = 0.10) for T2 EE of total PA: rho = 0.09 (*p* = 0.70) for T1, rho = -0.11 (*p* = 0.60) for T2 Walking slow: rho = -0.41 (*p* = 0.06) for T1, rho = 0.20 (*p* = 0.40) for T2 Walking fast: rho = 0.26 (*p* = 0.25) for T1, rho = 0.40 (*p* = 0.07) for T2 African Americans Sleep: rho = 0.22 (*p* = 0.09) for T1, rho = 0.55 (*p* < 0.0001) for T2 SB: rho = 0.06 (*p* = 0.70) for T1, rho = 0.36 (*p* = 0.008) for T2 Occupational/household LPA: rho = 0.11 (*p* = 0.40) for T1, rho = 0.17 (*p* = 0.20) for T2 Occupational/household MPA: rho = 0.03 (*p* = 0.80) for T1, rho = 0.30 (*p* = 0.02) for T2 Occupational/household VPA: rho = 0.15 (*p* = 0.30) for T1, rho = 0.56 (*p* < 0.0001) for T2 EE of sport/exercise: rho = 0.20 (*p* = 0.10) for T1, rho = 0.36 (*p* = 0.006) for T2 EE of total PA: rho = 0.08 (*p* = 0.50) for T1, rho = 0.15 (*p* = 0.30) for T2 Walking slow: rho = 0.26 (*p* = 0.05) for T1, rho = 0.27 (*p* = 0.04) for T2 Walking fast: rho = 0.35 (*p* = 0.007) for T1, rho = 0.36 (*p* = 0.005) for T2	Poor
Physical Activity Scale 2 (PAS 2) Pedersen et al., [101]	Danish version	Adults (mostly employed) *n* = 330 (203 female) age = 47 ± 9 years	Actiheart accelerometer-HR monitor (worn on chest)	Sleep + SB: PCC = 0.197 (*p* = 0.053); mean difference (PAS 2 - Acc) = -2.3 h/day; 95% LoA: -9.04 to 4.34 h/day LPA: PCC = 0.180 (*p* = 0.053); mean difference (PAS 2 - Acc) = 1.68 h/day; 95% LoA: 8.02 to -4.62 h/day MPA: PCC = 0.204 (*p* = 0.053); mean difference (PAS 2 - Acc) = 0.55 h/day; 95% LoA: 3.37 to -2.26 h/day VPA: PCC = 0.535 (*p* = 0.044); mean difference (PAS 2 - Acc) = 0.12 h/day; 95% LoA: 0.57 to 0.33 h/day	Good
Sedentary Time and Activity Reporting Questionnaire (STAR-Q) Csizmadi et al., [91]	English version	Adults (mostly employed) *n* = 99 Characteristics of baseline sample: *n* = 102 (61 female) age (male) = 51 ± 7 years age (female) = 46 ± 9 years	7-Day Activity Diary	EE of sleep: rho = 0.62 (*p* < 0.001); ICC = 0.18 (95% CI: -0.02, 0.38); median difference (STAR-Q - Diary) (IQR): -0.8 (0.8) h/day EE of SB: rho = 0.40 (*p* < 0.001); ICC = 0.12 (95% CI: -0.08, 0.31); median difference (STAR-Q - Diary) (IQR): -3.9 (4.9) h/day EE of LPA: rho = 0.26 (*p* = 0.009); ICC = 0.26 (95% CI: 0.07, 0.43); median difference (STAR-Q - Diary) (IQR): 0.6 (7.3) h/day EE of MPA: rho = 0.57 (*p* < 0.001); ICC = 0.49 (95% CI: 0.33, 0.63); median difference (STAR-Q - Diary) (IQR): 0.2 (5.0) h/day EE of VPA: rho = 0.68 (*p* < 0.001); ICC = 0.65 (95% CI: 0.52, 0.75); median difference (STAR-Q - Diary) (IQR): 0.0 (3.3) h/day	Fair
Simple Physical Activity Questionnaire (SIMPAQ) - modified (standing item was added) Schilling et al., [103]	German version	Young adults (university students) *n* = 72 (36 female) age = 23 ± 3 years	ActiGraph wGT3X-BT accelerometer (wrist worn)	Time in bed: rho = 0.35 (*p* < 0.01) SB: rho = 0.26 (*p* < 0.05) Standing (vs. Acc LPA): rho = 0.09 (*p* > 0.05) Walking (vs. Acc LPA): rho = 0.19 (*p* > 0.05) Non-structured PA (vs. Acc LPA): rho = − 0.2 (*p* > 0.05) Exercise (vs. Acc MVPA): rho = 0.49 (*p* < 0.001)	Good
24-hour movement behaviors questionnaire (24HMBQ) Zheng et al., [98]	Chinese version	Young adults (college students) *n* = 142 (37.3% female) age = 19.38 ± 2.53 years	PSQI, ASBQC, IPAQ-SF	Sleep: rho = 0.32 (*p* < 0.01); ICC = 0.40 (95% CI: 0.16, 0.57) SB: rho = 0.43 (*p* < 0.01); ICC = 0.61 (95% CI: 0.46, 0.72) Total PA: rho = 0.33 (*p* < 0.01); ICC = 0.57 (95% CI: 0.40, 0.69)	Poor
**Time-use recalls**					
7-Day Physical Activity Recall (7D PAR) - modified (sitting item was added) Welk et al., [97]	English version	Adults (physically inactive) *n* = 24 age: 38–57 years	Tritrac-R3D accelerometer (waist worn)	EE of rest (sleep + sitting): *r* = 0.27 (*p* > 0.05) EE of LPA (1.1–2.9 MET): *r* = 0.39 (*p* > 0.05) EE of MPA (3.0-4.9 MET): *r* = 0.47 (*p* < 0.05) EE of VPA (5.0-6.9 MET): *r* = 0.60 (*p* < 0.05) EE of VVPA (≥ 7 MET): *r* = 0.73 (*p* < 0.05) EE of MVPA (≥ 3MET): *r* = 0.53 (*p* < 0.05)	Poor
Computer-Based 24-Hour Physical Activity Recall (cpar24) Kohler et al., [96]	German version	Adults & older adults *n* = 49 (25 female) age = 50 ± 13 years	Accelerometer Actigraph GT3X+ (waist worn)	Sleep + SB: rho = 0.54; mean difference (cpar24 - Acc) = -31 min/day (*p* = 0.39); 95% LoA: -380 to 319 min/day LPA: rho = 0.46; mean difference (cpar24 - Acc) = -98 min/day (*p* < 0.001); 95% LoA: -399 to 204 min/day MVPA: rho = 0.50; mean difference (cpar24 - Acc) = 128 min/day (*p* < 0.001); 95% LoA: -151 to 407 min/day	Fair
**Time-use diary**					
Time-use diary from the Harmonised European Time Use Study (TUD HETUS) Harms et al., [92]	English version	Adults (mostly highly educated) *n* = 131 (approx. 60% female) age: 18 + years	Autographer wearable camera (on a lanyard or clipped to clothing)	Sleep, personal care: *r* = 0.852 (*p* < 0.0005) Eating and drinking: *r* = 0.550 (*p* < 0.0005) Paid work and related: *r* = 0.981 (*p* < 0.0005) Unpaid work, childcare: *r* = 0.924 (*p* < 0.0005) Voluntary/civic activity: *r* = 0.905 (*p* < 0.0005) Social activity, relaxation: *r* = 0.795 (*p* < 0.0005) Physical activity: *r* = 0.789 (*p* < 0.0005) Games, hobbies: *r* = 0.670 (*p* < 0.0005) TV, radio, read, IT: *r* = 0.653 (*p* < 0.0005) Travel: *r* = 0.861 (*p* < 0.0005)	Excellent

*Abbreviations:* Acc, accelerometer; CCC, concordance correlation coefficient; CI, confidence interval; ASBQC, Adult Sedentary Behaviors Questionnaire in China; EE, energy expenditure; ICC, intraclass corelation coefficient; IPAQ-SF, International Physical Activity Questionnaire - Short Form; LPA, light-intensity physical activity; LoA, limits of agreement; MPA, moderate-intensity physical activity; MVPA, moderate- to vigorous-intensity physical activity; PA, physical activity; PCC, polychoric correlation coefficient; *r*, Pearson correlation coefficient; PSQI, Pittsburgh Sleep Quality Index; rho, Spearman’s correlation coefficient; SB, sedentary behaviour; T1, administered at first occasion; T2, administered at second occasion; VPA, vigorous-intensity physical activity; VVPA, very vigorous-intensity physical activity

*Note:* Studies validated time spent in movement behaviours (in min/day), unless otherwise stated

Studies aggregated self-reported movement behaviours in a diversity of 24-h time-use compositions before being validated. Most studies validated daily time spent in sleep, SB, and physical activity of different intensities, one study validated domain-specific movement behaviours [90], and one study validated time spent in “super domains” [92]. All studies validated each component (i.e., aggregated self-reported movement behaviours) of 24-h time-use composition in isolation. For example, validity correlation coefficients for sleep time ranged between 0.22 and 0.69, for SB between 0.06 and 0.57, for LPA between 0.18 and 0.46, and for MVPA between 0.38 and 0.56.

### Reliability of self-reported methods

A total of 11 studies evaluated reliability of 10 unique self-reported tools for assessment of 24-h movement behaviours among adults (Table 3). Three studies were ranked with a good quality [91, 94, 98], seven with a fair quality [95, 96, 99, 100, 102, 104, 105], and one with a poor quality [90]. Studies differed substantially regarding time interval between test and retest administrations (ranged between > 4 h to 15 months). Four studies used Intraclass corelation coefficient to evaluate test-retest reliability, while seven studies reported only Pearson/Spearman’s correlation coefficient. Some studies also reported Bland-Altman statistics (e.g., mean difference, limits of agreement). All studies evaluated test-retest reliability of each component of 24-h time-use composition in isolation. For example, reliability correlation coefficients for sleep time ranged between 0.41 and 0.92, for SB between 0.33 and 0.91, for LPA between 0.55 and 0.94, and for MVPA between 0.59 and 0.94.

**Table 3:** Reliability of self-reported measurement tools

Title of the tool	Language	Sample characteristics	Time interval	Reliability indicators	Quality of the study
**Questionnaires**					
Daily Activity Behaviours Questionnaire (DABQ) Kastelic et al., [94]	Slovenian version	Adults (employed) *n* = 114 (46 female) age = 39 ± 8 years	1 week	Sleep: ICC = 0.59 (95% CI: 0.48, 0.69); rho = 0.64 (95% CI: 0.52, 0.74) SB: ICC = 0.65 (95% CI: 0.55, 0.73); rho = 0.62 (95% CI: 0.49, 0.72) LPA: ICC = 0.69 (95% CI: 0.60, 0.77); rho = 0.67 (95% CI: 0.56, 0.76) MVPA: ICC = 0.65 (95% CI: 0.55, 0.73); rho = 0.60 (95% CI: 0.47, 0.71)	Good
Japan Public Health Center-based prospective study- physical activity questionnaire (JPHC-PAQ) Kikucki et al., [95]	Japan version	Adults & older adults (mostly employed) *n* = 110 (57 female) age = 61 ± 6 years	3–6 months	Sleep: rho = 0.534 (*p* < 0.001) SB + LPA: rho = 0.708 (*p* < 0.001) MPA: rho = 0.483 (*p* < 0.001) VPA: rho = 0.745 (*p* < 0.001) MVPA: rho = 0.588 (*p* < 0.001)	Fair
Physical activity questionnaire (PAQ) Bharathi et al., [99]	English version	Adults (staff and students of the medical college) *n* = 112 (67 female) range: 18–60 years	2–4 weeks	EE of sleep: *r* = 0.91 (*p* < 0.01) EE of occupational sitting: *r* = 0.73 (*p* < 0.01) EE of occupational standing: *r* = 0.65 (*p* < 0.01) EE of occupational walking: *r* = 0.80 (*p* < 0.01) EE of occupational strenuous activities: *r* = 0.42 (*p* < 0.01) EE of leisure SB: *r* = 0.33 (*p* < 0.01) EE of hobbies: *r* = 0.62 (*p* < 0.01) EE of household chores: *r* = 0.85 (*p* < 0.01) EE of exercise: *r* = 0.71 (*p* < 0.01) “Residual” EE to 24 h: *r* = 0.50 (*p* < 0.01)	Fair
Physical Activity Questionnaire from the Southern Community Cohort Study (PAQ SCCS) Buchowski et al., [90]	English version	Adults *n* = 84–86 Characteristics of baseline sample (87 African American, 31 non-Hispanic whites): *n* = 118 (61 female) age = 55 ± 8 years	12–15 months	Sleep: rho = 0.41 (*p* < 0.0001) SB: rho = 0.33 (*p* = 0.002) Occupational/household LPA: rho = 0.08 (*p* = 0.48) Occupational/household MPA: rho = 0.06 (*p* = 0.55) Occupational/household VPA: rho = 0.04 (*p* = 0.70) Sport/Exercise MPA: rho = 0.36 (*p* = 0.0007) Sport/Exercise VPA: rho = 0.48 (*p* < 0.0001) Walking slow: rho = 0.24 (*p* = 0.03) Walking fast: rho = 0.26 (*p* = 0.01)	Poor
Physical Activity Scale 2.1 (PAS 2.1) - modified (transportation sitting item was added) Valles-Medina et al., [104]	Spanish version	Adults *n* = 51	3 weeks	Sleep: *r* = 0.82 (*p* < 0.001) Sitting at work/school hours: *r* = 0.363 (*p* = 0.009) Standing or walking at work/school hours: *r* = 0.791 (*p* < 0.001) Climbing stairs or carrying heavy objects at work/school hours: *r* = 0.718 (*p* < 0.001) Transportation sitting: *r* = 0.558 (*p* < 0.001) Leisure sitting while using screens/reading: *r* = 0.366 (*p* = 0.008) Leisure LPA: *r* = 0.744 (*p* < 0.001) Leisure MPA: *r* = 0.604 (*p* < 0.001) Leisure VPA: *r* = 0.880 (*p* < 0.001)	Fair
Sedentary Time and Activity Reporting Questionnaire (STAR-Q) Csizmadi et al., [91]	English version	Adults (mostly employed) *n* = 91–95 Characteristics of baseline sample: *n* = 102 (61 female) age (male) = 51 ± 7 years age (female) = 46 ± 9 years	3 months (T1 vs. T2); 6 months (T1 vs. T3)	STARQ1 vs. STARQ2 EE of sleep: ICC = 0.79 (95% CI: 0.70, 0.85) EE of SB: ICC = 0.53 (95% CI: 0.37, 0.66) EE of LPA: ICC = 0.60 (95% CI: 0.46, 0.71) EE of MPA: ICC = 0.45 (95% CI: 0.28, 0.60) EE of VPA: ICC = 0.65 (95% CI: 0.52, 0.75) STARQ1 vs. STARQ3 EE of sleep: ICC = 0.69 (95% CI: 0.57, 0.78) EE of SB: ICC = 0.45 (95% CI: 0.28, 0.59) EE of LPA: ICC = 0.55 (95% CI: 0.40, 0.67) EE of MPA: ICC = 0.51 (95% CI: 0.35, 0.64) EE of VPA: ICC = 0.52 (95% CI: 0.36, 0.65) STARQ1 vs. STARQ2 vs. STARQ3 EE of sleep: CCC = 0.73 (95% CI: 0.61, 0.81) EE of SB: CCC = 0.50 (95% CI: 0.40, 0.60) EE of LPA: CCC = 0.60 (95% CI: 0.45, 0.71) EE of MPA: CCC = 0.40 (95% CI: 0.29, 0.52) EE of VPA: CCC = 0.61 (95% CI: 0.48, 0.73)	Good
Simple Physical Activity Questionnaire (SIMPAQ) Rosenbaum et al., [102]	Multiple languages	Adults with mental illness (outpatients) *n* = 452 age: 18–65 years	1 week	Time in bed: rho = 0.75 (*p* < 0.001) SB: rho = 0.69 (*p* < 0.001) Walking: rho = 0.76 (*p* < 0.001) Exercise: rho = 0.76 (*p* < 0.001) Non-structured PA: rho = 0.63 (*p* < 0.001)	Fair
Simple Physical Activity Questionnaire (SIMPAQ) Vancampfort et al., [105]	Luganda version	Adults with mental illness (outpatients) *n* = 55 (34 female) age (female): 34 ± 8 years age (male): 38 ± 13 years	6 h	Time in bed: rho = 0.95 (*p* < 0.001) Napping: rho = 0.92 (*p* < 0.001) SB: rho = 0.91 (*p* < 0.001) Walking: rho = 0.96 (*p* < 0.001) Exercise: rho = 0.78 (*p* < 0.001) Non-structured PA: rho = 0.94 (*p* < 0.001)	Fair
24-hour movement behaviors questionnaire (24HMBQ) Zheng et al., [98]	Chinese version	Young adults (college students) *n* = 229 (51.5% female) age = 21.3 ± 2.52 years	1 week	Sleep (weekday): ICC = 0.80 (95% CI: 0.74, 0.84); rho = 0.64 (*p* < 0.01) Sleep (weekend): ICC = 0.74 (95% CI: 0.67, 0.80); rho = 0.65 (*p* < 0.01) SB (weekday): ICC = 0.80 (95% CI: 0.74, 0.85); rho = 0.68 (*p* < 0.01) SB (weekend): ICC = 0.65 (95% CI: 0.55, 0.73); rho = 0.51 (*p* < 0.01) Total PA (exercise): ICC = 0.80 (95% CI: 0.74, 0.85); rho = 0.69 (*p* < 0.01) Total PA (transport): ICC = 0.74 (95% CI: 0.66, 0.80); rho = 0.73 (*p* < 0.01) Total PA (dormitory life): ICC = 0.69 (95% CI: 0.59, 0.76); rho = 0.59 (*p* < 0.01)	Good
**Time-use recalls**					
Computer-Based 24-Hour Physical Activity Recall (cpar24) Kohler et al., [96]	German version	Adults & older adults *n* = 67 (34 females) age = 52 ± 13 years	Not reported	Sleep + SB: rho = 0.75; mean difference = -17 min/day (*p* = 0.60); 95% LoA: -292 to 259 min/day LPA: rho = 0.65; mean difference = 20 min/day (*p* = 0.89); 95% LoA: -256 to 296 min/day MVPA: rho = 0.92; mean difference = -3 min/day (*p* = 0.68); 95% LoA: -109 to + 102 min/day	Fair
Multimedia Activity Recall for Children and Adults (MARCA) Hunt et al., [100]	English version	Older adults with COPB and their carers *n* = 48 (24 female) age = 72 ± 10 years	> 4 h	Sleep: ICC = 0.92; mean difference = 7 min/day; 95% LoA: -83 to 98 min/day Total sitting time: ICC = 0.89; mean difference = -10 min/day; 95% LoA: -139 to 119 Sedentary (1-1.9 METs): ICC = 0.90; mean difference = -7 min/day; 95% LoA: -142 to 129 min/day Screen time: ICC = 0.94; mean difference = 5 min/day; 95% LoA: -91 to 161 min/day Light (2-2.9 METs): ICC = 0.94; mean difference = -2 min/day; 95% LoA: -79 to 76 min/day MVPA: ICC = 0.94; mean difference = 2 min/day; 95% LoA: -62 to 65 min/day Sport/exercise: ICC = 0.99; mean difference = 0 min/day; 95% LoA: -10 to 10 min/day	Fair

*Abbreviations*: CI, confidence interval; EE, energy expenditure; ICC, intraclass corelation coefficient; LPA, light-intensity physical activity; LoA, limits of agreement; MPA, moderate-intensity physical activity; MVPA, moderate- to vigorous-intensity physical activity; PA, physical activity; r, Pearson correlation coefficient; rho, Spearman’s correlation coefficient; SB, sedentary behaviour; T1, administered at first occasion; T2, administered at second occasion; T3, administered at third occasion; VPA, vigorous-intensity physical activity

*Note:* Studies validated time spent in movement behaviours (in min/day), unless otherwise stated

## Discussion

This systematic review identified 12 validated tools – eight questionnaires, three time-use recalls, and one time-use diary – for assessment of movement behaviours across the whole 24-h day. Most self-reported tools were designed for assessment of sleep, and domain-specific SB and physical activity, and generally showed adequate validity and/or reliability to be used in large-scale epidemiological studies and population surveillance.

Most self-reported tools included in our review showed comparable validity (DABQ, JPHC-PAQ, PAS 2, STAR-Q, 24HMBQ, 7D PAR, cpar24, TUD HETUS) and/or reliability (DABQ, JPHC-PAQ, PAQ, PAS 2.1, STAR-Q, SIMPAQ, 24HMBQ, cpar24, MARCA) correlation coefficients with the validity and/or reliability of most self-reported tools for assessment of a single movement behaviour [29–37]. The highest validity was observed for time-use diary TUD HETUS (*r* range: 0.55 to 0.92), and the highest test-retest reliability for short questionnaire SIMPAQ (rho range: 0.78 to 0.95) and computerised time-use recall MARCA (ICC range: 0.89–0.99). Higher validity of time-use diaries compared to other self-reports that rely on recalling more distant activities has been reported previously [106], and it was suggested that higher validity is associated with diminished recall bias. The highest reliability was observed in two studies that administered self-reported tool twice within the same day [100, 105], while the lowest reliability in a study where time interval between two administrations was more than one year [90]. However, it has been proposed that adequate time interval between two administrations is more than one day (to avoid recalling answers from the first administration), but less than three months for most tools (to guarantee sufficient stability of a behaviour *per se*) [107]. Therefore, reliability findings in these studies might differ if using adequate time intervals.

Self-reported time spent in sleep, SB, and physical activity is usually under- or over-estimated [29–37], which lead to sum of behaviours that do not equal to 24 h. However, most of the self-reported tools included in our review accounted that a sum of behaviours should add to 24 h by using different approaches. Some tools assigned the “remaining time to 24 hours” to either SB or LPA (DABQ, JPHC-PAQ, PAQ, PAS 2, STAR-Q, 7D PAR), one questionnaire (SIMPAQ) provided an alternative method for calculating SB by subtracting non-SB estimates from 24 h, two computerised recalls (cpar24, MARCA) ensured complete data entry by specific features of the program, and time-use diary (TUD HETUS) encourage responder to report activities during the 24-h period by providing a pre-defined recording fields. Most tools that used such approaches showed at least fair validity (DABQ, JPHC-PAQ, STAR-Q, 7D PAR, cpar24, TUD HETUS) and/or reliability (DABQ, JPHC-PAQ, PAQ, PAS 2.1, STAR-Q, SIMPAQ, cpar24, MARCA) for all movement behaviours examined. This is of great importance especially for studies that use compositional data analysis, since all components of time-use composition (e.g., 24-h movement behaviours composition consisting of time spent in sleep, SB, and physical activity) need to have adequate validity and/or reliability so that the time-use composition can be considered as valid and/or reliable.

By using COSMIN checklist, we found that only five validity studies (Table 2) and three reliability studies (Table 3) were ranked with at least good quality. The most frequent reasons for compromised quality of validity studies were poor selection of a reference measure, and insufficient sample size. The most frequent reasons for compromised quality of reliability studies were insufficient description of test and retest conditions, less appropriate use of statistical methods, less optimal time interval between two administrations, and a lack of description how missing data were handled. The COSMIN checklist (Supplementary Tables 2 and 3) can be used in future validation studies to guide methodological decisions in order to achieve high quality of the study. However, a careful consideration should be given to the choice of a reference measure since it is currently largely unknown which tools for assessment of 24-h movement behaviours could be considered as the best reference measure [108–111]. Accelerometers were frequently proposed to be a “reasonable gold standard” for assessment of free-living movement behaviours; however, hip placement is a preferred location for accurate assessment of physical activity [112], while thigh placement for SB [113], and wrist placement for sleep duration [114]. To avoid usage of multiple accelerometers, it was proposed that the best compromise might be using the same accelerometer at the hip during wake time and at the wrist during bedtime [115], or to combine thigh-worn accelerometer with sleep time diary [116]. The latest method was used in two studies included in our review [93, 94], while other studies used accelerometer placed on the chest, waist, or wrist [96, 97, 101, 103], wearable camera [92], or other self-reported method [90, 91, 95, 98].

Another important consideration is use of statistical analysis. Since data quantifying time spent in movement behaviours are compositional data, a recent study questioned the appropriateness of using Pearson/Spearman’s correlation coefficient and Intraclass correlation coefficients in studies examining validity and reliability of movement behaviours estimates [94]. Although those methods are recommended by COSMIN checklist, they are not intended for compositional data [7]. It has been warned that using traditional statistical methods (that were developed for data in real space) when dealing with compositional data (that lay in a constrained simplex space), may produce misleading results [9, 117]. To the best of our knowledge, compositional data analysis for evaluating validity and reliability of movement behaviours estimates are lacking. Therefore, to further support the development of 24-h movement behaviours research, there is a need to develop statistical analysis suitable for evaluation of validity and reliability of compositional data.

### Considerations for research, policy, and practice

The choice of the measurement tool depends on the objective of the study, measurement characteristics of a tool, and resources available. Several decision matrix guides to selecting physical activity or SB measurement tools have been described previously [23, 24, 27]. When selecting the tool, the first step is usually to identify which domains and dimensions of movement behaviours are of interest, and for what purpose data are collected (e.g., study design, individual level counselling). Then, a careful consideration regarding measurement characteristics of the tools (e.g., validity, reliability) and resources available (e.g., cost, time available for administration) is needed.

If the purpose is epidemiological research on the relationship between 24-h movement behaviours and health outcomes, then the important measurement characteristics are strong validity correlations and low random error [23]. However, if the purpose is to assess movement behaviours in longitudinal or intervention studies, then responsiveness to detect change is of great importance [24]. In our review, the strongest validity correlation coefficients were observed for the time-use diary TUD HETUS, while some other tools showed fair-to-substantial correlation coefficients (DABQ, JPHC-PAQ, STAR-Q, 24HMBQ, cpar24), which can be also deemed as sufficient for epidemiological research. However, TUD HETUS and cpar24 assesses behaviours during a single day, indicating that more than one day of assessment is needed to get a representative estimate of individual’s movement behaviours [25], which present additional burden. Therefore, DABQ, JPHC-PAQ, and STAR-Q may be better choice for adult population, and 24HMBQ for a specific population of dormitory students. Those four self-reports also showed fair-to-good reliability coefficients, while quality ratings for their validation studies were fair-to-excellent. None of the studies included in our review reported responsiveness, and therefore, no recommendations for longitudinal or intervention studies could be made.

If the purpose is population surveillance, then low systematic error and high responsiveness to detect change in behaviour of a population are important characteristics [23, 24]. Low systematic error is important for accurate assessment of the proportion of population that have (un)healthy pattern of movement behaviours, while responsiveness is needed to follow population trends. In population health surveys, there is usually a limited space available for questions on movement behaviours, indicating that shorter questionnaires (JPHC-PAQ, PAQ, PAS 2, SIMPAQ) may be more appropriate for most surveys. However, PAS 2 and SIMPAQ showed poor validity (*r* < 0.21 for some estimates), while only reliability has been evaluated for PAQ. Therefore, JPHC-PAQ might be a preferred choice. Among shorter questionnaires, Bland-Altman plot has been reported only for PAS 2; physical activity estimates were systematically higher, while sum of sleep and SB systematically lower when compared with the reference measure [101]. As the reference measure was not a reasonable gold standard, those findings could not be interpreted as measurement errors. Future studies should carefully consider choosing a highly trusted reference measure and exploring systematic and random error. Also, responsiveness of such tools to detect trends in a population behaviour is yet to be explored.

In practice, assessment of 24-h movement behaviours is usually needed for individual level estimates. If the purpose is to assess whether individual meet recommended levels of 24-h movement behaviours, then important measurement properties are high sensitivity and specificity for such classification [118]. If the purpose is to assess change in individual’s behaviour, then responsiveness to detect change on an individual level needs to be high [24]. As clinicians are usually interested in clinically meaningful change, minimal detectable change (i.e., change that is beyond normal within-individual variability in behaviour and the measurement error and can be interpreted as real change for an individual [24]) should be lower than minimal important change (i.e., minimal within-individual change above which individuals/patients perceive themselves importantly changed [119]). However, none of the studies included in our review explored sensitivity and specificity neither responsiveness to detect change on an individual level. Two studies on test-retest reliability [96, 100] and four studies on construct validity [93, 94, 96, 101] reported substantially large random error (e.g., 95% limits of agreement for MVPA estimate ranged from − 109 to + 102 min/day [96]), indicating that minimal detectable change for these self-reports is likely to be too large to detect minimal important change. In clinical care settings, there is usually only a limited time available for counselling on movement behaviours, and it has been recommended that tool need to be quick to administer (up to three minutes), and to provide immediate feedback [21]. Therefore, only short questionnaires (JPHC-PAQ, PAQ, PAS 2, SIMPAQ) may be a potential candidate tool. As mentioned above, PAQ, PAS 2, and SIMPAQ could not be recommended due to poor or unknown construct validity.

### Limitations

This review has some limitations that should be highlighted. First, the review was limited to studies that validated self-reported estimates of movement behaviours across the full 24-h day. This reduced the number of included studies, since studies that did not validate all components of the 24-h day were not included. According to the 24-h movement paradigm, movement behaviours are components of a finite total, and therefore, all components of the total need to be validated simultaneously. Therefore, self-reported tools evaluated in some excluded studies [64–83], need further validation of the whole 24-h time-use composition. Our review also did not include some important time-use tools used in the Multinational Time Use Study (MTUS) that harmonised over 100 national time-use surveys, and therefore, present a key resource for time-use research [120]. It might be that validation studies of most national time-use surveys are lacking. However, our review included TUD HETUS that showed high validity, and it might be that other similar time-use surveys have comparable validity. Second, literature search was conducted in only three databases, and therefore, we might miss some of the relevant studies. However, we used a comprehensive search query and conducted a secondary search, including citation searching, screening authors’ archive of references, and conducted a secondary database search on titles of identified self-reported tools. Third, most studies were conducted on convenience samples; therefore, findings might not be directly generalizable to the general population. As most tools were validated only in one language, future translation and cross-cultural validation studies might be needed for some self-reports.

## Conclusions

This systematic review identified 12 validated self-reported tools for assessment of 24-h movement behaviours, indicating that only a limited number of tools are currently available. Validation studies generally showed adequate construct validity and test-retest reliability to be used in epidemiological studies and population surveillance, while little is known about adequacy for individual level assessments and responsiveness to behavioural change. To better support research, policy, and practice on 24-h movement behaviours, there is a need for further developments in measurement methods. There is a need to develop new tools for assessment of 24-h movement behaviours for specific purposes and/or to adapt the existing physical activity and SB self-reports in a way that they will resonate with the emerging 24-h movement paradigm. Future studies should examine measurement properties of 24-h movement behaviours estimates simultaneously and by using statistical methods that respect compositional nature of movement behaviours data.

### Electronic supplementary material

Below is the link to the electronic supplementary material.

Supplementary Material 1

Supplementary Material 2

## Data Availability

All data generated or analysed during this study are included in this published article and its supplementary information file.

## Abbreviations

24HMBQ: 24-hour movement behaviors questionnaire
7D PAR: 7-Day Physical Activity Recall
COSMIN: Consensus-based Standards for the selection of health Measurement Instruments
CPAR24: Computer-Based 24-Hour Physical Activity Recall
DABQ: Daily Activity Behaviours Questionnaire
JPHC-PAQ: Japan Public Health Center-based prospective study- physical activity questionnaire
LPA: Light-intensity physical activity
MARCA: Multimedia Activity Recall for Children and Adults
MVPA: Moderate- to vigorous-intensity physical activity
PAQ: Physical activity questionnaire
PAQ SCCS: Physical Activity Questionnaire from the Southern Community Cohort Study
PAS 2: Physical Activity Scale 2
PAS 2.1: Physical Activity Scale 2.1
PRISMA: Preferred Reporting Items for Systematic Reviews and Meta-Analyses
PROSPERO: International Prospective Register of Systematic Reviews
SB: Sedentary behaviour
SIMPAQ: Simple Physical Activity Questionnaire
STAR-Q: Sedentary Time and Activity Reporting Questionnaire
TUD HETUS: Time-use diary from the Harmonised European Time Use Study
