# Supplementary material for: Validity and reliability of self-reported methods for assessment of 24-h movement behaviours: a systematic review
Source: Int J Behav Nutr Phys Act. 2024 Aug 2;21:83. doi: 10.1186/s12966-024-01632-4 (PMC11295502; doi:10.1186/s12966-024-01632-4)
Supplement: Supplementary file 2 — Supplementary Material 2 [file 12966_2024_1632_MOESM2_ESM.docx]

**SUPPLEMENTARY FILE**

**Supplementary Table 1.** Search strategy

| **Database** | **24-h Movement behaviours** | **Self-reported measures** | **Validity/reliability** |
| --- | --- | --- | --- |
| *PubMed* | (("physical activity"[Title/Abstract] OR "physical activities"[Title/Abstract]) AND (sedentary[Title/Abstract] OR sitting[Title/Abstract]) AND (sleep[Title/Abstract] OR sleeping[Title/Abstract] OR bedtime[Title/Abstract] OR "time in bed"[Title/Abstract] OR time-in-bed[Title/Abstract] OR rest[Title/Abstract]))  OR  (("time use"[Title/Abstract] OR time-use[Title/Abstract] OR "use of time"[Title/Abstract] OR use-of-time[Title/Abstract]) AND ("physical activity"[Title/Abstract] OR "physical activities"[Title/Abstract]))  OR  (("movement behaviour"[Title/Abstract] OR "movement behaviours"[Title/Abstract] OR "movement behavior"[Title/Abstract] OR "movement behaviors"[Title/Abstract] OR "physical behaviour"[Title/Abstract] OR "physical behaviours"[Title/Abstract] OR "physical behavior"[Title/Abstract] OR "physical behaviors"[Title/Abstract] OR "activity behaviour"[Title/Abstract] OR "activity behaviours"[Title/Abstract] OR "activity behavior"[Title/Abstract] OR "activity behaviors"[Title/Abstract] OR "24h activity"[Title/Abstract] OR "24 h activity"[Title/Abstract] OR "24-h activity"[Title/Abstract] OR "24h activities"[Title/Abstract] OR "24 h activities"[Title/Abstract] OR "24-h activities"[Title/Abstract] OR "24hour activity"[Title/Abstract] OR "24 hour activity"[Title/Abstract] OR "24-hour activity"[Title/Abstract] OR "24hour activities"[Title/Abstract] OR "24 hour activities"[Title/Abstract] OR "24-hour activities"[Title/Abstract] OR "24hours activity"[Title/Abstract] OR "24 hours activity"[Title/Abstract] OR "24-hours activity"[Title/Abstract] OR "24hours activities"[Title/Abstract] OR "24 hours activities"[Title/Abstract] OR "24-hours activities"[Title/Abstract] OR "24h physical activity"[Title/Abstract] OR "24 h physical activity"[Title/Abstract] OR "24-h physical activity"[Title/Abstract] OR "24h physical activities"[Title/Abstract] OR "24 h physical activities"[Title/Abstract] OR "24-h physical activities"[Title/Abstract] OR "24hour physical activity"[Title/Abstract] OR "24 hour physical activity"[Title/Abstract] OR "24-hour physical activity"[Title/Abstract] OR "24hour physical activities"[Title/Abstract] OR "24 hour physical activities"[Title/Abstract] OR "24-hour physical activities"[Title/Abstract] OR "24hours physical activity"[Title/Abstract] OR "24 hours physical activity"[Title/Abstract] OR "24-hours physical activity"[Title/Abstract] OR "24hours physical activities"[Title/Abstract] OR "24 hours physical activities"[Title/Abstract] OR "24-hours physical activities"[Title/Abstract]) AND ("physical activity"[Title/Abstract] OR "physical activities"[Title/Abstract])) | questionnaire*[Title/Abstract] OR recall*[Title/Abstract] OR diary[Title/Abstract] OR diaries[Title/Abstract] OR ediary[Title/Abstract] OR e-diary[Title/Abstract] OR self report*[Title/Abstract] OR self-report*[Title/Abstract] OR proxy report*[Title/Abstract] OR proxy-report*[Title/Abstract] OR "ecological momentary assessment"[Title/Abstract] OR EMA[Title/Abstract] OR survey*[Title/Abstract] OR surveillance[Title/Abstract] OR tool*[Title/Abstract] OR instrument*[Title/Abstract] OR measur*[Title/Abstract] OR interview*[Title/Abstract] OR "assessment method"[Title/Abstract] OR "assessment methods"[Title/Abstract] OR "subjective method"[Title/Abstract] OR "subjective methods"[Title/Abstract] OR log[Title/Abstract] OR logs[Title/Abstract] | valid*[Title/Abstract] OR reliab*[Title/Abstract] OR test-retest[Title/Abstract] OR responsive*[Title/Abstract] OR comparison[Title/Abstract] OR comparative[Title/Abstract] OR comparable[Title/Abstract] OR psychometric*[Title/Abstract] OR concordance[Title/Abstract] OR reproducib*[Title/Abstract] OR accur*[Title/Abstract] OR precis*[Title/Abstract] OR repeatab*[Title/Abstract] OR replica*[Title/Abstract] OR agreement[Title/Abstract] OR sensitiv*[Title/Abstract] OR specificity[Title/Abstract] |
| *Scopus* | TITLE-ABS-KEY ( ( ( "physical activity" OR "physical activities" ) AND ( sedentary OR sitting ) AND ( sleep OR sleeping OR bedtime OR "time in bed" OR time-in-bed OR rest ) ) )  OR  TITLE-ABS-KEY ( ( ( "time use" OR time-use OR "use of time" OR use-of-time ) AND ( "physical activity" OR "physical activities" ) ) )  OR  TITLE-ABS-KEY ( ( ( "movement behaviour" OR "movement behaviours" OR "movement behavior" OR "movement behaviors" OR "physical behaviour" OR "physical behaviours" OR "physical behavior" OR "physical behaviors" OR "activity behaviour" OR "activity behaviours" OR "activity behavior" OR "activity behaviors" OR "24h activity" OR "24 h activity" OR "24-h activity" OR "24h activities" OR "24 h activities" OR "24-h activities" OR "24hour activity" OR "24 hour activity" OR "24-hour activity" OR "24hour activities" OR "24 hour activities" OR "24-hour activities" OR "24hours activity" OR "24 hours activity" OR "24-hours activity" OR "24hours activities" OR "24 hours activities" OR "24-hours activities" OR "24h physical activity" OR "24 h physical activity" OR "24-h physical activity" OR "24h physical activities" OR "24 h physical activities" OR "24-h physical activities" OR "24hour physical activity" OR "24 hour physical activity" OR "24-hour physical activity" OR "24hour physical activities" OR "24 hour physical activities" OR "24-hour physical activities" OR "24hours physical activity" OR "24 hours physical activity" OR "24-hours physical activity" OR "24hours physical activities" OR "24 hours physical activities" OR "24-hours physical activities" ) AND ( "physical activity" OR "physical activities" ) ) ) | TITLE-ABS-KEY ( ( questionnaire* OR recall* OR diary OR diaries OR ediary OR e-diary OR self AND report* OR self-report* OR proxy AND report* OR proxy-report* OR "ecological momentary assessment" OR ema OR survey* OR surveillance OR tool* OR instrument* OR measur* OR interview* OR "assessment method" OR "assessment methods" OR "subjective method" OR "subjective methods" OR log OR logs ) ) | TITLE-ABS-KEY ( ( valid* OR reliab* OR test-retest OR responsive* OR comparison OR comparative OR comparable OR psychometric* OR concordance OR reproducib* OR accur* OR precis* OR repeatab* OR replica* OR responsive* OR agreement OR sensitiv* OR specificity ) ) |
| *SPORTDiscus (through EBSCO)* | TI ( (("physical activity" OR "physical activities") AND (sedentary OR sitting) AND (sleep OR sleeping OR bedtime OR "time in bed" OR time-in-bed OR rest))  OR  (("time use" OR time-use OR "use of time" OR use-of-time) AND ("physical activity" OR "physical activities"))  OR  (("movement behaviour" OR "movement behaviours" OR "movement behavior" OR "movement behaviors" OR "physical behaviour" OR "physical behaviours" OR "physical behavior" OR "physical behaviors" OR "activity behaviour" OR "activity behaviours" OR "activity behavior" OR "activity behaviors" OR "24h activity" OR "24 h activity" OR "24-h activity" OR "24h activities" OR "24 h activities" OR "24-h activities" OR "24hour activity" OR "24 hour activity" OR "24-hour activity" OR "24hour activities" OR "24 hour activities" OR "24-hour activities" OR "24hours activity" OR "24 hours activity" OR "24-hours activity" OR "24hours activities" OR "24 hours activities" OR "24-hours activities" OR "24h physical activity" OR "24 h physical activity" OR "24-h physical activity" OR "24h physical activities" OR "24 h physical activities" OR "24-h physical activities" OR "24hour physical activity" OR "24 hour physical activity" OR "24-hour physical activity" OR "24hour physical activities" OR "24 hour physical activities" OR "24-hour physical activities" OR "24hours physical activity" OR "24 hours physical activity" OR "24-hours physical activity" OR "24hours physical activities" OR "24 hours physical activities" OR "24-hours physical activities") AND ("physical activity" OR "physical activities")) )  OR  AB ( (("physical activity" OR "physical activities") AND (sedentary OR sitting) AND (sleep OR sleeping OR bedtime OR "time in bed" OR time-in-bed OR rest))  OR  (("time use" OR time-use OR "use of time" OR use-of-time) AND ("physical activity" OR "physical activities"))  OR  (("movement behaviour" OR "movement behaviours" OR "movement behavior" OR "movement behaviors" OR "physical behaviour" OR "physical behaviours" OR "physical behavior" OR "physical behaviors" OR "activity behaviour" OR "activity behaviours" OR "activity behavior" OR "activity behaviors" OR "24h activity" OR "24 h activity" OR "24-h activity" OR "24h activities" OR "24 h activities" OR "24-h activities" OR "24hour activity" OR "24 hour activity" OR "24-hour activity" OR "24hour activities" OR "24 hour activities" OR "24-hour activities" OR "24hours activity" OR "24 hours activity" OR "24-hours activity" OR "24hours activities" OR "24 hours activities" OR "24-hours activities" OR "24h physical activity" OR "24 h physical activity" OR "24-h physical activity" OR "24h physical activities" OR "24 h physical activities" OR "24-h physical activities" OR "24hour physical activity" OR "24 hour physical activity" OR "24-hour physical activity" OR "24hour physical activities" OR "24 hour physical activities" OR "24-hour physical activities" OR "24hours physical activity" OR "24 hours physical activity" OR "24-hours physical activity" OR "24hours physical activities" OR "24 hours physical activities" OR "24-hours physical activities") AND ("physical activity" OR "physical activities")) )  OR  KW ( (("physical activity" OR "physical activities") AND (sedentary OR sitting) AND (sleep OR sleeping OR bedtime OR "time in bed" OR time-in-bed OR rest))  OR  (("time use" OR time-use OR "use of time" OR use-of-time) AND ("physical activity" OR "physical activities"))  OR  (("movement behaviour" OR "movement behaviours" OR "movement behavior" OR "movement behaviors" OR "physical behaviour" OR "physical behaviours" OR "physical behavior" OR "physical behaviors" OR "activity behaviour" OR "activity behaviours" OR "activity behavior" OR "activity behaviors" OR "24h activity" OR "24 h activity" OR "24-h activity" OR "24h activities" OR "24 h activities" OR "24-h activities" OR "24hour activity" OR "24 hour activity" OR "24-hour activity" OR "24hour activities" OR "24 hour activities" OR "24-hour activities" OR "24hours activity" OR "24 hours activity" OR "24-hours activity" OR "24hours activities" OR "24 hours activities" OR "24-hours activities" OR "24h physical activity" OR "24 h physical activity" OR "24-h physical activity" OR "24h physical activities" OR "24 h physical activities" OR "24-h physical activities" OR "24hour physical activity" OR "24 hour physical activity" OR "24-hour physical activity" OR "24hour physical activities" OR "24 hour physical activities" OR "24-hour physical activities" OR "24hours physical activity" OR "24 hours physical activity" OR "24-hours physical activity" OR "24hours physical activities" OR "24 hours physical activities" OR "24-hours physical activities") AND ("physical activity" OR "physical activities")) ) | TI (questionnaire* OR recall* OR diary OR diaries OR ediary OR e-diary OR self report* OR self-report* OR proxy report* OR proxy-report* OR "ecological momentary assessment" OR EMA OR survey* OR surveillance OR tool* OR instrument* OR measur* OR interview* OR "assessment method" OR "assessment methods" OR "subjective method" OR "subjective methods" OR log OR logs)  OR  AB (questionnaire* OR recall* OR diary OR diaries OR ediary OR e-diary OR self report* OR self-report* OR proxy report* OR proxy-report* OR "ecological momentary assessment" OR EMA OR survey* OR surveillance OR tool* OR instrument* OR measur* OR interview* OR "assessment method" OR "assessment methods" OR "subjective method" OR "subjective methods" OR log OR logs)  OR  KW (questionnaire* OR recall* OR diary OR diaries OR ediary OR e-diary OR self report* OR self-report* OR proxy report* OR proxy-report* OR "ecological momentary assessment" OR EMA OR survey* OR surveillance OR tool* OR instrument* OR measur* OR interview* OR "assessment method" OR "assessment methods" OR "subjective method" OR "subjective methods" OR log OR logs) | TI (valid* OR reliab* OR test-retest OR responsive* OR comparison OR comparative OR comparable OR psychometric* OR concordance OR reproducib* OR accur* OR precis* OR repeatab* OR replica* OR responsive* OR agreement OR sensitiv* OR specificity)  OR  AB (valid* OR reliab* OR test-retest OR responsive* OR comparison OR comparative OR comparable OR psychometric* OR concordance OR reproducib* OR accur* OR precis* OR repeatab* OR replica* OR responsive* OR agreement OR sensitiv* OR specificity)  OR  KW (valid* OR reliab* OR test-retest OR responsive* OR comparison OR comparative OR comparable OR psychometric* OR concordance OR reproducib* OR accur* OR precis* OR repeatab* OR replica* OR responsive* OR agreement OR sensitiv* OR specificity) |

**Supplementary Table 2.** Assessing the quality of studies examining the construct validity.

|  | **Excellent (= 3)** | **Good (= 2)** | **Fair (= 1)** | **Poor (= 0)** |
| --- | --- | --- | --- | --- |
| **Design requirements** |  |  |  |  |
| Was the percentage of missing items given? | Percentage of missing items described | Percentage of missing items NOT described |  |  |
| Was there a description of how missing items were handled? | Described how missing items were handled | Not described but it can be deduced how missing items were handled | Not clear how missing items were handled |  |
| Was the sample size included in the analysis adequate? | Adequate sample size (≥100) | Good sample size (50-99) | Moderate sample size (30-49) | Small sample size (<30) |
| Is it clear what the comparator instrument(s) measure(s)? | Constructs measured by the  comparator instrument(s) is  clear |  | Constructs measured by the  comparator instrument(s) is  not clear |  |
| Were the measurement properties of the comparator  instrument(s) sufficient? | Sufficient measurement  properties of the comparator  instrument(s) in a population similar to the study population | Sufficient measurement  properties of the comparator  instrument(s) but not sure if these apply to the study population | Some information on measurement properties of the  comparator instrument(s) in any study population | No information on the measurement properties of the  comparator instrument(s), OR  evidence for insufficient measurement properties of the  comparator instrument(s) |
| Were there any important flaws in the design or methods of the study? | No other important methodological flaws |  | Other minor methodological flaws | Other important methodological flaws in the design or execution of the study |
| **Statistical methods** |  |  |  |  |
| For continuous scores: Were correlations, or the area under the receiver operating curve calculated? | Correlations or AUC calculated |  |  | Correlations or AUC NOT calculated |
| For dichotomous scores: Were sensitivity and specificity determined? | Sensitivity and specificity calculated |  |  | Sensitivity and specificity NOT calculated |

Table adapted from Terwee C. B., et al. Rating the methodological quality in systematic reviews of studies on measurement properties: a scoring system for the COSMIN checklist. Quality of Life Research. 2012.

* we defined a ‘sufficient’ measurement property of the comparator instrument(s) if showed a correlation ≥ 0.5 with another superior comparator instrument(s) (adapted from: Terwee et al. Qualitative Attributes and Measurement Properties of Physical Activity Questionnaires: A Checklist. 2020.)

**Supplementary Table 3.** Assessing the quality of studies examining the reliability.

|  | **Excellent (= 3)** | **Good (= 2)** | **Fair (= 1)** | **Poor (= 0)** |
| --- | --- | --- | --- | --- |
| **Design requirements** |  |  |  |  |
| Was the percentage of missing items given? | Percentage of missing items described | Percentage of missing items NOT described |  |  |
| Was there a description of how missing items were handled? | Described how missing items were handled | Not described but it can be deduced how missing items were handled | Not clear how missing items were handled |  |
| Was the sample size included in the analysis adequate? | Adequate sample size (≥100) | Good sample size (50-99) | Moderate sample size (30-49) | Small sample size (<30) |
| Were at least two measurements available? | At least two measurements |  |  | Only one measurement |
| Were the administrations independent? | Independent measurements | Assumable that the measurements were independent | Doubtful whether the measurements were independent | measurements NOT independent |
| Was the time interval stated? | Time interval stated |  | Time interval NOT stated |  |
| Were patients stable in the interim period on the construct to be measured? | Patients were stable (evidence provided) | Assumable that patients were stable | Unclear if patients were stable | Patients were NOT stable |
| Was the time interval appropriate?* | Time interval appropriate |  | Doubtful whether time interval was appropriate | Time interval NOT appropriate |
| Were the test conditions similar for both measurements? e.g. type of administration, environment, instructions | Test conditions were similar (evidence provided) | Assumable that test conditions were similar | Unclear if test conditions were similar | Test conditions were NOT similar |
| Were there any important flaws in the design or methods of the study? | No other important methodological flaws in the design or execution of the study |  | Other minor methodological flaws in the design or execution of the study | Other important methodological flaws in the design or execution of the study |
| **Statistical methods** |  |  |  |  |
| For continuous scores: Was an intraclass correlation coefficient (ICC) calculated? | ICC calculated and model or formula of the ICC is described | ICC calculated but model or formula of the ICC not described or not optimal.  Pearson or Spearman correlation coefficient calculated with evidence provided that no systematic change has occurred | Pearson or Spearman correlation coefficient calculated WITHOUT evidence provided that no systematic change has occurred or WITH evidence that systematic change has occurred | No ICC or Pearson or Spearman correlations calculated |
| For dichotomous/nominal/ordinal scores: Was kappa calculated? | Kappa calculated |  |  | Only percentage agreement calculated |
| For ordinal scores: Was a weighted kappa calculated? | Weighted Kappa calculated |  | Unweighted Kappa calculated | Only percentage agreement calculated |
| For ordinal scores: Was the weighting scheme described? e.g. linear, quadratic | Weighting scheme described | Weighting scheme NOT described |  |  |

Table adopted from Terwee C. B., et al. Rating the methodological quality in systematic reviews of studies on measurement properties: a scoring system for the COSMIN checklist. Quality of Life Research. 2012.

* we defined an ‘adequate’ time interval between test and re-test as follows: > 1 day and ≤ 3 months for questionnaires recalling a usual week/month; > 1 day and ≤ 2 weeks for questionnaires recalling the previous week; > 1 day and ≤ 1 week for questionnaires recalling the previous day; > 1 day and ≤ 1 year for questionnaires recalling the previous year (adopted from: Sattler et al. Current Evidence of Measurement Properties of Physical Activity Questionnaires for Older Adults: An Updated Systematic Review. Sports Medicine. 2020.)
